# Suboptimal asthma care for immigrant children: results of an audit study

**DOI:** 10.1186/1472-6963-8-22

**Published:** 2008-01-24

**Authors:** JJ Nathalie Urbanus-van Laar, Johan S de Koning, Niek S Klazinga, Karien Stronks

**Affiliations:** 1 Department of Social Medicine, Academic Medical Center-University of Amsterdam, The Netherlands; 2 National Institute for Public Health and the Environment, Bilthoven, The Netherlands

## Abstract

**Background:**

Little is known on the scope and nature of ethnic inequalities in suboptimal asthma care for children. This study aimed to assess (1) ethnic differences in suboptimal asthma care for children with an asthma exacerbation who consulted a physician, and (2) ethnic differences in the nature of suboptimal care.

**Methods:**

All children aged 6–16 years who during a period of six months consulted the paediatric department of the Academic Medical Centre-University of Amsterdam or one of the six regional primary care centres with an asthma exacerbation were included. Clinical guidelines were systematically converted to review criteria following the strategy as proposed by the Agency for Health Care Policy and Research. Based upon these review criteria and their experience experts of two multidisciplinary panels retrospectively assessed the quality of care and its (possible) failure to prevent the occurrence of asthma exacerbation.

**Results:**

Only a small number of children (n = 35) were included in the analysis as a result of which the ethnic differences in suboptimal care were not significant. However, the results do indicate immigrant children, in particular 'other non-Western' children (n = 11), more frequently to receive suboptimal care related to the asthma exacerbation when compared to ethnic Dutch children. Furthermore, we found the nature of suboptimal care to differ with under-prescribing in the 'other non-Western' group (n = 11), lack of information exchange between physicians in the Surinamese/Antillean group (n = 12) and lack of education, and counselling of patients and parents in the ethnic Dutch (n = 12) as the most relevant factor.

**Conclusion:**

Ethnic inequalities in the scope and nature of suboptimal asthma care for children in the Netherlands seem to exist. For the non-western immigrant groups the results indicate the importance of the prescription behaviour of the medical doctor, as well as the supervision by one health care provider.

## Background

Asthma is one of the most common chronic diseases and its prevalence has increased markedly during the past few decades in Western Europe. This places a large burden on patients and health care. It is estimated, for example, that about 15 million disability-adjusted life years are lost per year due to asthma, and that asthma accounts for about 1 of each 250 deaths worldwide [1]. In children, asthma accounts for many lost school days which may result in deprivation of educational achievement and social interaction [2].

Studies from the USA and the UK found immigrants to be disproportionately affected by asthma. Immigrant children were found to have higher asthma related mortality rates and hospitalizations rates than their peers [1,3,4]. For other Western countries, information on ethnic inequalities in asthma related mortality and morbidity is scant.

Though often put forward as a potential explanation [5,6], little evidence exists on the relation between inadequate asthma care and ethnic inequalities in asthma morbidity. Studies, mostly from the USA and the UK, showed quality of asthma care for immigrant children to be poorer than their White counterparts. These studies found immigrant children to receive less often anti-inflammatory medicines, β2 agonists, and timely follow-up after ED visit [7-12]. These studies assessed some limited aspects of asthma care, while adequate asthma care depends on a conjunction of a variety of factors, such as asthma medications, routine follow-ups, education and counseling, and self-management. Moreover, the extent to which these ethnic inequalities in asthma care contribute to the relatively poor health of children in immigrant groups is unknown. More insight should be obtained in ethnic differences in the nature of suboptimal asthma care and its relationship with asthma morbidity, as this may give specific indications for quality improvement.

In our aim to identify opportunities for improvement, we appraised the quality of asthma care provided in Amsterdam, the Netherlands by performing an audit among children who consulted a physician because of an asthma exacerbation. A clinical audit into avoidable factors, which may influence adverse outcomes like asthma exacerbation, allows us to identify shortcomings in the process of care in relation to this adverse outcome [13].

This study aimed to assess (1) ethnic differences in suboptimal asthma care for children with an asthma exacerbation who consulted a physician, and (2) ethnic differences in the nature of suboptimal care. In the Netherlands and other European countries a variety of health care providers can be involved in the care for children with asthma [14]. We therefore decided to review the asthma care delivered by all providers concerned (general practitioners, paediatricians and paediatric lung specialists) in the two years preceding the asthma exacerbation. Details on the care delivered were presented to a multidisciplinary expert panel that retrospectively assessed the quality of care and its (possible) failure to prevent the occurrence of asthma exacerbation.

## Methods

### Design

We performed an audit of asthma care provided two years preceding an asthma exacerbation. The six primary care centres in Amsterdam Southeast and the paediatric department of the Academic Medical Centre-University of Amsterdam all participated in the audit. On the basis of review criteria data on processes of care were collected by extracting data from medical records and by means of structured interviews. Subsequently, two panels of experts assessed (possible) shortcomings in asthma care and its relation to the occurrence of the asthma exacerbation. These panels were both multidisciplinary and each consisted of a general practitioner (GP), a paediatrician, a paediatric lung specialist, and a physician assistant or a nurse-practitioner.

### Patients

All children aged 6–16 years who, during the period December 2002 till June 2003, consulted one of the six primary healthcare centres in Amsterdam Southeast or the paediatric department of the Academic Medical Centre-University of Amsterdam with an asthma exacerbation, were selected. These children all needed to have a physician diagnosis of asthma. A child was considered to have an asthma exacerbation when it consulted a physician outside scheduled visits because of shortness of breath.

Three strategies were used to identify children with exacerbation of asthma. First, all contacts with the primary care centres by children aged 6–16 years were checked for symptoms which may indicate at respiratory problems (complaints of shortness of breath, exacerbation, moaning or dyspnoea). We asked the GP if the child indeed had consulted him because of an asthma exacerbation. Personal data of the patients, including ethnicity, were anonymous to the researchers.

Second, the children selected via the Academic Medical Centre-University of Amsterdam were presented by their physician and patients and parents were asked informed consent. To ensure we would not miss children with an exacerbation of asthma we regularly approached several key figures and asked them if they knew of new children. Amongst these key figures were all three Paediatric lung specialists, two senior pediatricians at the outpatient clinic and the physician-assistant at the outpatient clinic. Third, for the Academic Medical Centre-University of Amsterdam we have manually checked all ED- forms in the period December 2002 till June 2003 and screened the forms on date of birth and reason for ED visit. Subsequently, we checked with the key figures whether the children we thought to be eligible for the study indeed contacted the ED department because of an exacerbation of asthma.

**Figure 1:**
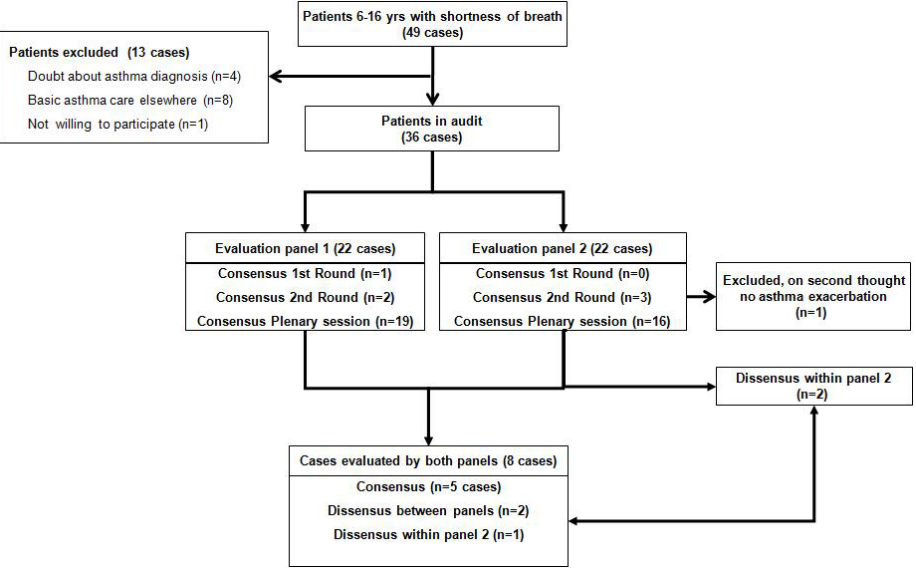
Patient selection, the distribution of cases between the panels and the results of the assessment of asthma care.

In total 36 children participated in the study (Figure 1).

### Review criteria

Three practice guidelines on asthma care for children [15-17], all meant to be used in 2000–2003, were systematically converted to review criteria following the strategy as proposed by the Agency for Health Care Policy and Research (AHCPR) [18]. The most relevant domains within the guidelines were identified and the guidelines were subsequently converted to review criteria along established lines.

In total, 57 criteria were formulated, covering the following areas: (1) education and counselling, (2) prescriptions and types of inhalation devices, (3) routine follow-ups, (4) referrals and shared care, (5) acute care. During a plenary session with experts in the field of asthma care for children the relevance and applicability of these criteria were assessed and minor adjustments were made accordingly.

### Data collection

Based upon the formulated review criteria a questionnaire was constructed in such a way it was possible to collect data on the formulated review criteria. In addition, data was gathered on patient characteristics and medical history regarding provoking factors and severity of complaints. This so the experts could judge the asthma care provided two years before asthma exacerbation taking asthma severity, asthmatic recurrences and provoking factors into account. With this questionnaire a research assistant and the corresponding author collected information by extracting data from medical records and by means of structured interviews with physicians for data which could not be extracted. Patient characteristics extracted were age, gender and ethnic background. Ethnicity was determined on the basis of country of origin of the parents according to the definition of Statistics Netherlands (CBS) [19]. For the children selected via the Academic Medical Centre-University of Amsterdam parents were asked for their countries of birth, while for children who were selected through GPs the GP informed us on the countries of births of the parents.

The majority of the immigrant children in the Netherlands originate from non-Western countries. Within Amsterdam Southeast the majority of children are of Surinamese and Antillean origin- a former colony of the Netherlands and six islands in the Caribbean Sea which are part of the Netherlands. In addition, a large number of children come from a variety of other non-Western countries with both children of labour migrants and refugees.

### Assessment of suboptimal care

We decided to spread the cases over two expert panels as the judgment of the cases was considered to be time-consuming (30–45 min per case). Each panel consisted of 4 members: a general practitioner, a paediatrician, a paediatric pulmonologist and a physician assistant or nurse-practitioner. These experts were selected based on their clinical expertise with respect to asthma care for children. To assess the reliability of the judgment procedure 8 cases were judged by both expert panels. This left 22 cases to be judged by both expert panels (Figure 1).

For each case they had to judge, the experts received a case description based on the questionnaire, as well as a copy of the questionnaire. In addition, suggestions were given regarding aspects of the delivered care which likely did not meet the 57 formulated review criteria. Furthermore, the experts received the list with review criteria. They did not, however, receive information on the ethnic background of the cases. Based on identified factors of care, which did not meet the review criteria, responsible actors (physician, patient or health facility), severity of the identified suboptimal factors (minor or major) and its estimated relationship with the occurrence of the asthma exacerbation, the experts allocated a score on a scale of 0 to 3 to the delivered care, reflecting no suboptimal care present, suboptimal care present which unlikely, possibly or likely failed to prevent the asthma exacerbation respectively. With respect to non-compliance by the patient a decision rule was phrased: When the physician had done everything in his or her ability to promote compliance the score assigned to the case should be relatively low, e.g. 0 or 1 depending on other suboptimal factors identified. If the physician's efforts were not sufficient, the score should be relatively high, e.g. 2 or 3.

The quality of asthma care was evaluated in 3 rounds. In the first round, the experts independently assessed the quality of care delivered and its potential association with the occurrence of the exacerbation of asthma. In their judgment of the cases the experts could well-argued deviate from the review criteria.

In the second round, the experts were informed on the opinions of the other experts of the panel and again were asked to independently assess the quality of asthma care and its potential association with the occurrence of the exacerbation of asthma. Cases for which there was no agreement after two evaluation rounds were discussed within each panel. Figure 1 describes the patient selection, the distribution of cases between the panels and the results of the assessment of asthma care.

### Analysis

Characteristics of ethnic groups were described using proportions and medians with interquartile range (IQR), and analyzed with χ^2^-test and Kruskal-Wallis test. Ethnic differences in the suboptimal care were assessed with χ^2^-test. To gain more power for analysis, the grades of (sub)optimal care were dichotomized: a score of 0 indicating at care which unlikely failed to prevent the asthma exacerbation, and a score of 1 indicating at suboptimal care which possibly or even likely failed to prevent the asthma exacerbation. The suboptimal factors, which were considered by the experts to be the main arguments for suboptimal care delivered, were described as proportions of all identified suboptimal factors per ethnic group.

Analyses were performed using SPSS (release 12.0) (SPSS Inc. Chicago, USA).

## Results

A total of 36 cases were eligible to be included in the audit. One case was excluded from the analysis as, on second thought, panel 2 considered the child not to have had an asthma exacerbation. The experts agreed in all cases on the presence or absence of suboptimal factors. Four cases were not assigned a score because the experts disagreed on the relation of suboptimal care with the occurrence of the exacerbation of asthma (n = 3) or in the assessment whether the physician had done everything he could have done to improve compliance (n = 1). One of these cases was part of the reliability check which left 7 cases eligible for assessing the agreement between the two panels of experts. In 5 of these cases the experts agreed on the presence of suboptimal care in relation to the occurrence of the asthma exacerbation.

We had a relatively high number of children of Surinamese/Antillean background which reflects the ethnic composition of Amsterdam Southeast. The group with children of other ethnic groups consists of children from non-Western countries with the exception of one Australian child. These non-Western children originate from a variety of countries namely Morocco (2), India (2), Somalia (1), Turkey (1), Egypt (1), Sudan (1), Pakistan (1) and Dominicans Republic (1). We further refer to this group as the 'other non-Western' group.

Though not statistically significant, we found indications of immigrant children more often to have moderate asthma symptoms, while ethnic Dutch children either had mild or persistent symptoms. Furthermore, one quarter of the immigrant children received combined supervision of a GP and a specialist, while ethnic Dutch children were either under supervision of their GP or specialist. In Table 1 the characteristics of the children are described.

**Table 1:** Ethnic differences in the characteristics of the children

**Characteristics**	**Total **(n = 35)	**ethnic Dutch **(n = 12)	**Surinamese/Antillean **(n = 12)	**'Other non-western' **(n = 11)	**χ^2 ^(p-value)**
Age in years* (n = 35)	10 (8–12)	10.5 (6.25–14.25)	10.5 (8.25–13.00)	9 (6–10)	3.182 (0.204)
**Gender (n = 35)**					3.914 (0.141)
Female	51.4%	58.3%	66.7%	27.3%	
Male	48.6%	41.7%	33.3%	72.7%	
**Severity of symptoms (n = 29)**					1.824 (0.768)
Mild	44.8%	55.6%	41.7%	37.5%	
Intermittent	27.6%	11.1%	33.3%	37.5%	
Persistent	27.6%	33.3%	25.0%	25.0%	
**Health professionals (n = 35)**					4.996 (0.288)
GP	51.4%	66.7%	41.7%	45.5%	
GP & specialist	17.1%	0.0%	33.3%	18.2%	
Specialist	31.4%	33.3%	25.0%	36.4%	

* Medians with Interquartile Range, analyzed with Kruskal-Wallis test

Ethnic differences in receipt of suboptimal care in relation to the occurrence of the exacerbation of asthma are presented in Table 2. Of all children almost two third received care which possibly or likely had failed to prevent the asthma exacerbation. In particular 'other non-Western' children received suboptimal care which possibly or likely had failed to prevent the asthma exacerbation, though the differences were not statistically significant. When looking at the original allocated score on a scale of 0 to 3 a similar trend in ethnic differences in the receipt of suboptimal care was observed. The 'other non-Western' group more often scored suboptimal care which likely had failed to prevent the asthma exacerbation, the ethnic Dutch children more often scored no suboptimal care or suboptimal care which unlikely was related to the asthma exacerbation, while the Surinamese/Antillean group took a middle position (Data not shown).

**Table 2:** Ethnic differences in identified suboptimal asthma care

**Characteristics**	**Total **(n = 31)	**ethnic Dutch **(n = 11)	**Surinamese/Antillean **(n = 10)	**'Other non-western' **(n = 10)	**χ^2 ^(p)**
**Score**					2.645 (0.266)
No suboptimal care/Care unlikely associated with exacerbation	38.7%	54.5%	40.0%	20.0%	
Possibly or likely associated with exacerbation	61.3%	45.5%	60.0%	80.0%	

The nature of suboptimal care identified by the experts is presented in Table 3. A total of 49 suboptimal factors related to asthma exacerbation were identified for the 19 cases which received suboptimal care which possibly or likely had failed to prevent the asthma exacerbation. Suboptimal care regarding prescriptions, in particular undertreatment, was most frequently observed in our population. When looking at ethnic differences in the identified nature of suboptimal care, we found the 'other non-Western' group to have the highest number of suboptimal factors, with inadequate prescriptions as the most relevant suboptimal factor in this group. In the Surinamese/Antillean group the most relevant factor concerned problems in shared care which meant that for these children information exchange between physicians was often lacking; their medical records were poorly maintained and that they relatively frequently received care by a variety of physicians. The most relevant suboptimal factor in ethnic Dutch children was a lack of sufficient education and counselling of patients and parents.

**Table 3:** Ethnic differences in number and types of suboptimal factors for children who received suboptimal care which was possibly or likely related to the exacerbation

**Suboptimal factors N (%)**	**All patients**	**ethnic Dutch**	**Surinamese/Antillean**	**'Other non-western'**
Education & Counselling	11 (22.45)	**5 (33.33)**	3 (20.00)	3 (15,79)
Prescriptions & types of inhalation devices	**13 (26.53)**	4 (26.67)	2 (13.33)	**7 (36,84)**
Routine follow-ups	9 (18.37)	3 (20.00)	4 (26.67)	2 (10,53)
Referrals and shared care*	10 (20.41)	2 (13.33)	**5 (33.33)**	3 (15,79)
Patient Compliance & effort undertaken to promote compliance	4 (8.16)	1 (6.67)	1 (6.67)	2 (10,53)
Other	2 (4.08)	-	-	2 (10,53)

Total number of factors (%)	49 (100.0)	15 (30.61)	15 (30.61)	**19 (38,78)**

* This includes undesirably receiving care by a variety of physicians, lack of information exchange between physicians and poorly maintained records

In bold the most relevant suboptimal factors per ethnic group are given.

## Discussion

In this study we have assessed ethnic inequalities in suboptimal asthma care in relation to the occurrence of an asthma exacerbation, and ethnic differences in the nature of suboptimal care delivered. We found that approximately two third of the children received care which possibly or likely had failed to prevent the asthma exacerbation, and that this in particular seems to apply to 'other non-Western' children. Furthermore, we found indications for ethnic differences in the nature of suboptimal asthma care with under-prescribing in the 'other non-Western' group, problems in shared care in the Surinamese/Antillean group and lack of education, and counselling of patients and parents in the ethnic Dutch as the most relevant factor.

Before discussing the results some potential limitations of this study should be acknowledged. First, there is no generally accepted definition of asthma exacerbation [20]. In this study we defined children to have had exacerbation in asthma when a child consulted a physician outside scheduled visits. As a result of this definition we may have missed children who were able to control the asthma exacerbation by raising intake of relievers such as short-acting β2-agonists and did not consult a physician. On the other hand, some children in this study may have consulted a physician for complaints which were not that severe and who should have been able to control the complaints themselves at home. Yet, as the experts took the severity of asthma complaints and exacerbation into account when evaluating the quality of care in relation to the occurrence of the asthma exacerbation, we do not expect that this has influenced the judgments of the experts, nor do we expect that this has influenced the observed ethnic inequalities.

A second potential limitation concerns the determination of ethnicity. We asked the parents of children who were selected via the Academic Medical Centre-University of Amsterdam for their countries of birth, while for children who were selected through GPs the GP had to inform us on the countries of births of the parents. Judgement of countries of birth by others than patients or their parents themselves may lead to mistakes in the determination of ethnicity. Thus, some of the children who were presented by their GPs may have been miscategorised. However, as GPs work with registered patients and are likely to be able to distinguish 'other non-Western' children from children from Surinamese/Antillean or ethnic Dutch origin, we do not expect that this has led to systematic biases in our results.

Thirdly, only a small number of children were included as a result of which the ethnic differences in suboptimal care were not significant. As we have screened all contacts with the primary care centres, all ED-forms for the study period and regularly contacted key figures, we do not believe that we have systematically missed children nor do we think that we have missed children of certain ethnic groups. All things considered, we do not think that the direction of our findings would have changed substantially had more children been included in the analyses. These findings do suggest that immigrant children, in particular 'other non-Western' children, were more likely than ethnic Dutch children to receive suboptimal care which fails to prevent exacerbation in asthma. However, further study is needed to confirm or refute our findings.

Lastly, there are some limitations related to the design. Retrospective audits based on practice guidelines may encounter problems such as incomplete medical records, dated guidelines and difficulties in fully establishing a causal relationship between the quality of the delivered care and the observed adverse outcome. In this study we have tried to overcome these problems, by conducting structured interviews with physicians for data which could not be extracted from medical records. This, however, could not prevent that in particular for the Surinamese/Antillean group the medical records were poorly maintained. The expert panels considered the absence of information to be a potential threat for the quality of care, in particular because this absence of information seemed to occur in combination with combined supervision by GP and specialist. In this study incomplete medical records, therefore have not led to uncertainties in the assessment of the quality of the delivered care.

In addition, we have discussed new insights into adequate asthma care within a plenary session and combined expert opinion with evidence based practice guidelines and with new insights to determine the relationship between suboptimal asthma care and the occurrence of asthma exacerbation. More importantly, these two points are not likely to differ with the ethnic background of the children, and thus do not detract from our findings regarding the observed ethnic inequalities.

To our knowledge, no other studies to date have evaluated ethnic inequalities in asthma care related to the occurrence of exacerbation of asthma. Thus, the indication found in our study that immigrant children are more likely to have received suboptimal care which failed to prevent an asthma exacerbation is novel. This is particularly of interest when taken into account that immigrant children are found to have a higher burden of asthma both within as outside the Netherlands [1,3,4,21].

The findings of our study support others regarding ethnic inequalities in some limited aspects of asthma treatment [7-12]. The results of our study are also in line with a study in adults [22]. Similar to that study, we found patients who were supervised by their GP or received combined GP/specialist supervision more often to receive suboptimal care which possibly or likely had failed to prevent the exacerbation of asthma compared to children who were exclusively supervised by a specialist (70.6% and 75.0% versus 40.0%-Data not shown). In this study, only immigrant children, in particular Surinamese/Antillean children, received combined GP/specialist supervision. When taken into account that the most relevant suboptimal factor in the Surinamese/Antillean group concerns the coordination of care, these findings suggest that supervision by one health care provider is preferred above combined supervision.

The results of this study also indicate the nature of suboptimal care to differ by ethnicity. Undertreatment of the other non-Western group was most frequently mentioned by the experts as part of the care which failed to prevent the asthma exacerbation. Undertreatment was also found among Blacks and Hispanics in the US [9-12] and among children of the Indian subcontinent living in the UK [13]. Furthermore, immigrant children were found to receive less often care with the same provider than their peers [23], as found within the Surinamese/Antillean children within this study, while interpersonal continuity of care is likely to improve health [24].

Several factors related to the patient-physician interaction may explain the observed ethnic inequalities. Though we are not able to provide conclusive evidence, even with the small numbers within this study a clear trend could be observed: The more likely cultural distance and language barriers are present in consultations between physicians and patients, the more likely suboptimal care was present which failed to prevent the asthma exacerbation. Because of their territorial bond with the Netherlands, Surinamese/Antillean children and their parents are probably more likely to be acquainted with the Dutch culture and language than 'other non-Western' groups.

Our idea that language barriers and cultural distance are likely to influence the quality of care is supported by a Dutch study on shared decision making. In this study indications of problems in information exchange due to language barriers, cultural differences and bias or stereotyping were found [25]. In another Dutch study it was observed that in consultations with non-Western adults Dutch GP's were less often patient-centered and information was less often exchanged than in consultations with Dutch adults [26]. Furthermore, Van Ryn and Burke found physicians' perceptions to be affected by patient race and socio-economic status. According to their study: "Patient race was associated with physicians' assessment of patient intelligence, feelings of affiliation towards the patient, and beliefs about patient's likelihood of risk behavior and adherence with medical advice" [27].

These problems in the patient-physician encounter may have led to less adequate assessment of the severity of asthma complaints and needs. Moreover, it may have led to inadequate educating and counseling of patients, which in turn is likely to lead to difficulties in compliance. In some cases within this study the care was found to be insufficient which at first hand may have been judged as non-compliance by the patient and parents. Yet, in most of these cases the experts considered the physicians efforts not to have been sufficient to promote compliance. In their view, home visits by and consultations with special trained nurse practitioners or physician-assistants could have made a difference.

The findings of this study deserve attention as two third of the children in this study received care which possibly or likely had failed to prevent the asthma exacerbation. Particularly a trend was observed in which ethnic groups in which language barriers and cultural distance are more likely to be present than in ethnic Dutch children were more likely to receive suboptimal asthma care. Moreover, this study suggests that priorities for quality improvement should differ per ethnic group, though all suboptimal factors deserve attention.

Effective dissemination and feedback of results are necessities for encouraging quality improvement. As part of the audit we are currently in the process of informing the participating health care providers on the findings of this study. They at least should be aware of the observed ethnic inequalities in asthma care, and should examine reasons for poor performance. Room for quality improvement in particular is found in the care delivered by GPs or when combined GP/specialist supervision takes place. The question rises whether supervision by one health care provider should be preferred above combined supervision, both generally as in particular in immigrant children. Moreover, as language barriers and cultural distance are likely to play an important role in the observed ethnic inequalities in asthma care integrating special trained nurses or intercultural mediators and improving physicians' cultural competences may help in overcoming ethnic differences in health care quality.

## Conclusion

The findings of this study suggest that non-Western immigrant children are more likely to receive suboptimal asthma care in comparison to ethnic Dutch children. Furthermore, the nature of suboptimal care is also likely to differ by ethnicity

For the non-western immigrant groups the results indicate the importance of the prescription behaviour of the medical doctor, as well as the supervision by one health care provider.

## Competing interests

The author(s) declare that they have no competing interests.

## Authors' contributions

All the authors contributed to this study. JJNL, NSK and KS conceived of the study, JJNL participated in its design, collected the data, and performed the analyses, and drafted the manuscript. JSK, NSK and KS participated in the design of the study and in the data collection and refined the draft manuscript.

## Pre-publication history

The pre-publication history for this paper can be accessed here:


